# Nanofluidic devices prepared by an atomic force microscopy-based single-scratch approach

**DOI:** 10.1039/c9ra06428a

**Published:** 2019-11-27

**Authors:** Yongda Yan, Jiqiang Wang, Shunyu Chang, Yanquan Geng, Leyi Chen, Yang Gan

**Affiliations:** Key Laboratory of Micro-systems and Micro-structures Manufacturing of Ministry of Education, Harbin Institute of Technology Harbin Heilongjiang 150001 P. R. China gengyanquan@hit.edu.cn +86-451-86415244 +86-451-86412924; Center for Precision Engineering, Harbin Institute of Technology Harbin Heilongjiang 150001 P. R. China; School of Chemistry and Chemical Engineering, Harbin Institute of Technology Harbin 150001 China

## Abstract

Nanofluidic chips with different numbers of nanochannels were fabricated based on a commercial AFM system using a single-scratch approach. The electrical characterization and enzymatic reactions at the nanoscale were demonstrated using the obtained chips. The effects of the number of nanochannels and the solution concentration on the measured electric current were investigated. The influence of the hydrodynamic convection generated from the induced inflow at the end of the nanochannel on the ion transport through the nanochannel was also studied. Moreover, the enzymatic reactions for trypsin towards poly-l-lysine (PLL) or thrombin were conducted with a nanofluidic chip to investigate the reaction specificity between trypsin and PLL. Results show that the electric current change during the experimental process could be used as a label-free indicator to detect the enzymatic activity.

## Abstract 2

A nanofluidic chip was prepared based on a commercial AFM system. Effects on ion transport and enzymatic reaction specificity were demonstrated.

## Graphical abstract

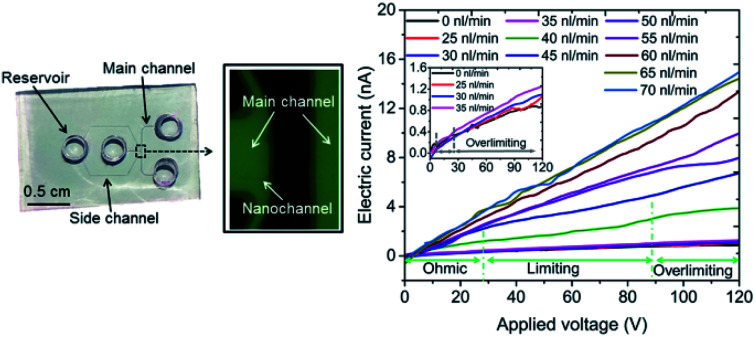

## Introduction

Nanofluidic devices have drawn significant attention because of their potential application in various fields, such as health care,^1^ medicine,^2^ and DNA/protein transport.^3,4^ Fabrication of nanofluidic chips using a simple and highly efficient approach has thus become a research focus and challenge for the application of nanofluidic devices. In addition, a number of physical phenomena, different to those observed with microchannels, have been observed in nanofluidic devices.^5,6^ For understanding their application, it is therefore critical to investigate, for example, the semiconducting properties of the electrolytes in nanochannels using a nanofluidic device fabricated by a low cost, simple, and highly efficient method.

Several fabricating approaches, such as reactive ion etching (RIE),^7^ photolithography,^8^ focus ion beam lithography (FIB),^9^ electron beam lithography (EBL),^10^ have thus far been used to prepare nanofluidic chips. All of these approaches are limited by shortcomings.^11,12^ For example, it is inconvenient for photolithography approach to change the photomasks when fabricate different micro/nanostructures. For FIB and EBL methods, the fabrication cost is extremely high, and a clean high vacuum environment is needed. However, one nanofabrication method, atomic force microscopy (AFM), has proven to be a feasible and powerful approach to machine nanostructures.^13–15^ Furthermore, tip-based nanofabrication, especially single scratch type processes, have specific advantages, including being a simple machining process and providing high fabricating efficiency. Hence, the nanochannels of the nanofluidic chips prepared in this work were fabricated using a single scratch approach.

Nanofluidic chips can be used to study ion transport characterization in nanochannels. Compared with microchannels, the surface to volume ratio of a nanochannel is larger. Hence, the electrokinetic phenomena are more vital in nanofluidic devices than in microfluidics.^16,17^ In addition, the ion concentration polarization (ICP) phenomenon, which refers to an imbalance of electrolyte concentrations nearby nanostructures under a DC bias, has the ability to control the charged ion in solution.^18^ The formation of ICP is affected by ion selectivity, which means counterions can pass across the nanostructures. In contrast, most of the co-ions fail to transport through nanostructures.^19^ The ICP commonly formed at the end of nanostructure adions would deplete at the anodic side and enrich at the cathodic side of the nanostructures.^20^ Thus, the electrical resistance for the ICP region increases from the lower ion concentration at the depletion zone. The electric current in the nanofluidic chips always shows an overlimiting characterization before a traditional ohmic–limiting region. The limiting current is regarded as a nuisance because it limits the transport of the ion in the nanochannel.^21^ For example, in the energy-harvesting systems such as fuel cell, it is desirable to obtain a higher power level at the same power input by eliminating the effect of the limiting current. Hence, it is necessary to obtain a high current with high electrical power efficiency when the applied voltage is not large enough to eliminate limiting current. To date, several approaches have been used to deal with this problem.^19,22,23^ However, they all used a permanent structure, which means tuning of the ohmic–limiting–overlimiting characteristics is impossible. A new method, therefore, is necessary to easily tune the ohmic–limiting–overlimiting current.

Recently, nanofluidic chips have been applied to label-free detection. The label-free technique, as one detection method, has great potential for bioengineering.^24^ Thus far, most label-free detection techniques have depended on surface reactions.^25,26^ Nevertheless, surface reactions manifest several limitations, such as lower reaction rates and difficulty in detecting ultralow concentrations. Label-free detection using nanofluidic devices can overcome this problem because the analytes are confined within a nanoscale space. Researchers have carried out several experiments to investigate proteolysis and enzyme kinetics based on this approach.^27,28^ However, the electrical signal generated from the reactions in the nanochannels was ignored, despite this being a potential indicator of the reaction process and enzyme activity.

Therefore, in this study, nanofluidic chips with single or multiple nanochannels were prepared based on a commercial AFM system using the single scratching approach. Furthermore, the effects of the number of nanochannels and electrolyte solution (KCl) concentration on the characterization of the measured electric current were investigated. To eliminate the influence of the overlimiting current, a hydrodynamic convection, generated from an outlet inflow, was induced into the end of the nanochannels. The enzymatic reaction specificity of trypsin towards poly-l-lysine (PLL) was proven based on a prepared nanofluidic chip by monitoring the characterization of the electric current during the experimental process.

## Experimental details

### Fabrication of nanochannel on a polycarbonate surface using AFM

**Fig. 1:**
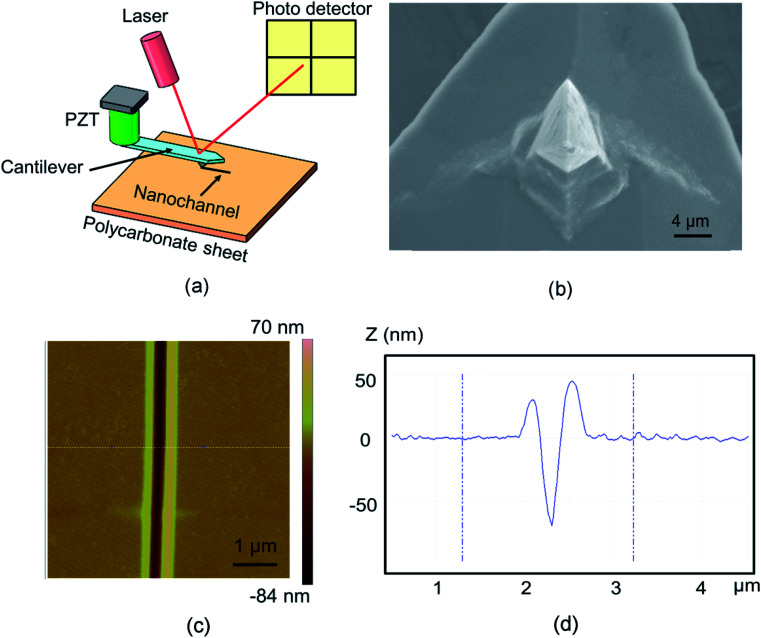
(a) Schematic diagram of the nanochannel fabrication, (b) SEM micrograph of diamond coated AFM tip, (c) AFM image, and (d) the cross-section of the nanochannel fabricated with a normal load of 20 μN and velocity of 3 μm s^−1^.

The fabrication processes for the studied nanochannels were carried out on a commercial AFM system (Dimension Icon, Bruker Company, Karlsruhe, Germany) under atmospheric conditions. A schematic diagram of the fabrication process using the single scratching approach is presented in Fig. 1(a). The AFM system used the Nanoman module and the tip approached the sample surface with a preset normal load. The normal load changed from 20 μN to 60 μN with a spacing of 10 μN. Furthermore, the machining velocity was selected as 3 μm s^−1^ for all scratching experiments. The probe was controlled to scratch along the side-forward direction. As shown in Fig. 1(b), a rectangular pyramidal tip with a 100 nm thick diamond coating (DT-NCLR, Nanosensors, Switzerland) was employed to perform the scratching process. The cantilever, which the tip is mounted on, was made of silicon with a normal spring constant (*K*_N_) of 68 N m^−1^ as provided by the manufacturer. All the topographies of the obtained structures were measured using a silicon tip (radius of 10 nm; TESPA, Bruker Company, Germany) by a tapping mode. An injection-molded PC sheet (Goodfellow, Huntingdon, England) with a molecular weight of 35 000 was used as the sample material. The mean and the standard deviation of the surface roughness (*R*_a_) of the PC sheet were measured as 0.6 nm and 0.2 nm, respectively, which were measured by scanning a 50 μm × 50 μm area of the sample using the AFM tapping mode. Fig. 1(c) and (d) present the AFM image and cross-section of the nanochannel fabricated with a normal load of 20 μN and velocity of 3 μm s^−1^. The width and depth of the obtained nanochannel are 200 and 60 nm, respectively.

### Preparation of the nanofluidic chips

Preparation of the nanofluidic chips consisted of fabrication of microchannels/nanochannels, transfer printing of these microchannels/nanochannels, and a chip bonding process. A convex microchannel mold on a silicon wafer surface was fabricated using UV photolithography, and the final concave microchannel was obtained after being transferred by PDMS (Sylgard 184, Dow Corning, USA). The nanochannel mold transfer consisted of two transfer steps. The convex wall was obtained by the first transfer process based on the nanochannel mold on the PC surface. Then, the convex wall worked as the mold for the second transfer process, and the concave nanochannel was prepared after this transfer using PDMS. The weight ratios of monomer to curing agent for PDMS during the first and second transfer processes were selected as 5 : 1 and 7 : 1, respectively. The prepared nanofluidic chips, which consisted of PDMS slabs with microchannels and nanochannels, were bonded by oxygen-plasma treatment (Zepto, Diener electronic, Germany) for a duration of 32 s under a chamber pressure of 1.5 mbar and a chamber power of 81 W. The enclosed micro/nanochannel chip were then bonded at a temperature of 95 °C for 20 min. More details on the transfer and bonding processes can be found in our previous study.^29^

### Preparation of the chemicals and protein solution

To investigate the effect of solution concentration on the conductivity of the nanochannels, different concentrations of KCl buffer solutions (0.1 mM, 1 mM, 10 mM, and 100 mM) were used in this study. In addition, the trypsin–PLL reaction was used as a prototypical example to demonstrate the electrical detection of enzymatic reactions in nanochannels. Trypsin plays an important role in the digestive system and takes part in the trypsin proteolysis process that cleaves peptide chains at the carboxyl side of the amino acids lysine and arginine. Trypsin powder (BioFroxx, Germany), and a concentration of 10^5^ ng mL^−1^ trypsin in buffer solution was prepared in this work. The concentrations for the PLL (Biosharp, Germany, molecular weight 70–150 kDa) and thrombin (Biosharp, Germany) solutions used in this work were 100 mg mL^−1^ and 10^5^ ng mL^−1^, respectively.

## Results and discussion

### Conductivity of nanofluidic chips using KCl solution

**Fig. 2:**
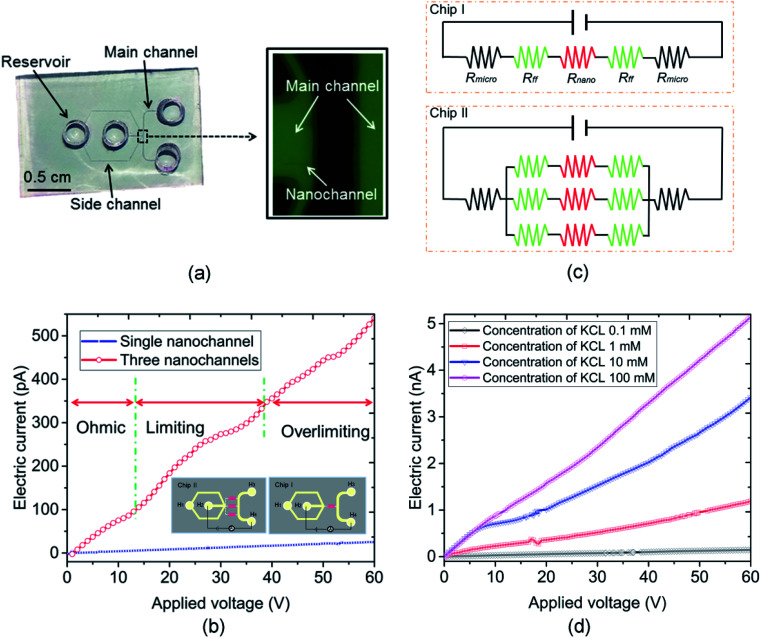
(a) Schematic diagram of nanofluidic chip, (b) electric current measurement results of chips I and II, (c) an equivalent electrical circuit of chips I and II that comprised of resistors, and (d) electric current measurement results for concentration of KCl from 0.1 to 100 mM.

In this work, the nanofluidic chips that contain one and three nanochannels are termed chip I and II, respectively. Fig. 2(a) presents a schematic diagram of chip I. The diameter of the reservoir was 2 mm, and the widths of the side and main channel were 15 and 80 μm, respectively. The distance between the two main channels was 30 μm. The concentration of the KCl solution used was 0.1 mM. The feature sizes of the nanochannels for nanofluidic chip I and II are identical to each other, with a depth and width of 29 and 170 nm, respectively. Fig. 2(b) shows the electric current measurement results for chips I and II. A schematic diagram of the measurement for electric current is present as the inset of Fig. 2(b). The current measurement experiments were carried out on an electrometer (Model 6430, Keithley, USA). For the *I*–*V* measurement a step voltage of 0.5 V was ramped every 2 s from 0 to 60 V by the software KickStart 2. It is noticeable in Fig. 2(b) that the electrical currents increase with the increase in the applied voltages. Furthermore, the current in chip II was higher than that in chip I. The current in chip II is about 500 pA; however, for chip I it is about 50 pA when the voltage reaches 60 V. The conductance in a nanochannel can be estimated by the following equation:^30^1
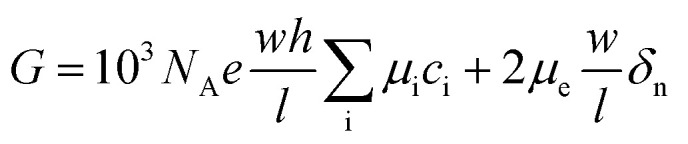
where *μ*_i_ is the mobility of ion i, *c*_i_ is the concentration of ion i, and *δ*_n_ is the effective surface charge inside the nanochannel. *N*_A_ and *e* signify Avogadro's constant and the electron charge, respectively. *w*, *h*, and *l* are the nanochannel width, height, and length, respectively. The width and depth of the nanochannel for chip I equal those for chip II. The charge density of a surface is a material property,^31^ thus *δ*_n_ in all channels is identical. According to eqn (1), the conductance for each nanochannel in chips I and II are the same, which means the resistance for the nanochannels is consistent. The diffuse layer thickness of electric double layer (EDL), which scales inversely to the bulk concentration, was found as 30–50 nm when the concentration of KCl solution is 1 mM.^32^ In the present study, the depth of the nanochannel and the KCL concentration were 29 nm and 0.1 mM, respectively, which means the top and bottom EDLs were overlapped in the nanofluidic system. Thus, the current nanochannel system is ideal permselectivity due to the completely overlapping of the EDLs. At low concentrations, the effect of microchannel and field focusing resistors should not be neglected for a three-layered micro–nano–microchannel system.^33^Fig. 2(c) shows the equivalent electrical circuits of chips I and II that comprised of resistors. The overall resistances for chips I (*R*_I_) and II (*R*_II_) are given by eqn (2) and (3), respectively.

2 *R*_I_ = 2*R*_micro_ + 2*R*_ff_ + *R*_nano_

3
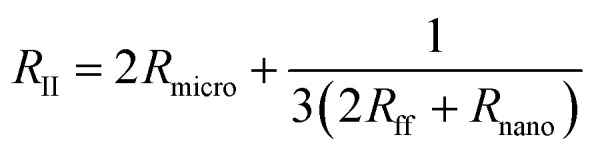
where *R*_micro_, *R*_ff_ and *R*_nano_ are the resistance of the microchannel, the fields focusing resistor and the nanochannel, respectively. The values of them are calculated as follows:^33^4
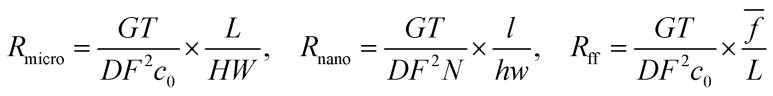
where *G* is the universal gas constant, *T* is the absolute temperature, *F* is the Faraday constant, and *D* is the diffusion coefficient. *c*_0_ and *N* denote the unstirred bulk concentration and the average excess counterion concentration within the nanochannels, respectively. The *L*, *H* and *W* represent for the length, height and width of the microchannel, respectively. It can be observed in eqn (4) that the resistances are depended on the structure size of the micro and nanochannels. The feature dimensions of the microchannel and nanochannel for chip I are identical with chip II, thus, the resistance of the microchannel and the fields focusing resistor for chips I and II are constant. In this condition, the overall resistance for chips II (*R*_II_) is smaller than I (*R*_I_) according to the eqn (2) and (3). Hence, the electric current in chip II, as shown in Fig. 2(b), is therefore much higher than that in chip I when the same voltage is applied. Furthermore, ohmic–limiting–overlimiting behavior^34,35^ can be observed in Fig. 2(b). When the applied voltage is lower than 14 V, the ion current increases almost linearly with the applied voltage increasing. When the applied voltage increasing, a limiting region appears due to the local ion concentration on the anodic side approaches zero, where the current is not increase linearly with the voltage increasing. However, when the applied voltage increasing further, the relationship between the electric current and applied voltage become linear again, which is called the overlimiting region.

Fig. 2(d) presents the electric current measurement results for KCl concentrations from 0.1 to 100 mM in a single channel nanofluidic chip (chip I). The width and depth of the nanochannel in this chip are 248 and 19 nm, respectively. It is noticeable from Fig. 2(d) that the electric current increases with the increase in the applied voltage. In addition, the largest electric current was measured at a concentration of 100 mM. The conductance of a single nanochannel can be estimated by eqn (1). It is observable from eqn (1) that the conductance depends on the feature size of the nanochannel, the ion concentration of solution, and the effective surface charge. In this study, the feature sizes of the nanochannels in the nanofluidic chip were consistent. At high ionic concentrations, the second term of eqn (1) (surface term) has a small influence on the total conductance, and the conductance is governed by the channel geometry and the bulk concentration.^36^ Thus, the electric current for a concentration of 100 mM was the highest when applying the same voltage.

**Fig. 3:**
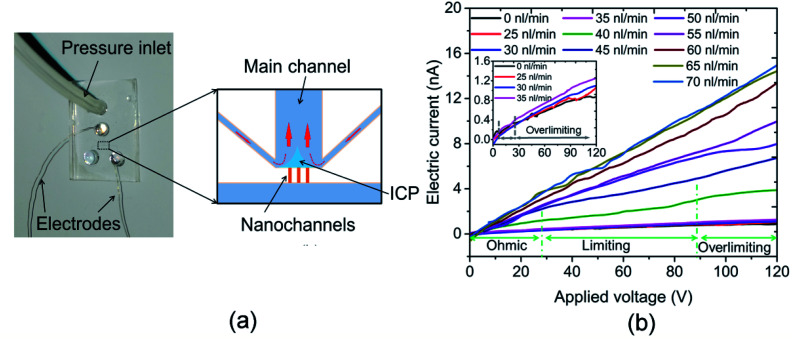
(a) Schematic diagram of the flow in a nanofluidic chip and (b) electric current measurement results for hydrodynamic inflow of KCl from 0 to 70 nL min^−1^. The inset display the measurement results for hydrodynamic inflow from 0 to 35 nL min^−1^.

ICP refers to an imbalance of electrolyte concentrations near nanostructures under a DC bias. The formation of the ICP is affected by ion selectivity, and the ion selectivity means counterions can pass across the nanostructures, such as a nanochannel or nanoporous membrane. In contrast, most of the co-ions fail to transport through the nanostructures. The characterization of an ICP field can be described by (1) the ion concentration inside the ICP field is lower than that outside, and (2) the area of the ICP field is unstable. The resistance of the depletion zone for the ICP field is relatively high due to the lower ion concentration. Thus, an overlimiting current followed by a traditional ohmic–limiting region is initiated as the transport mechanism. The limiting current in the nanochannel is regarded as a nuisance for the application of nanofluidic chips because it limits the transport of the ion in the nanochannel. Thus, a hydrodynamic convection at the end of the nanochannel induced by an inflow was used to decrease the effect of the limiting current. The nanofluidic device with three nanochannels (chip II) was used in this experiment, and the width and depth of the nanochannel were 144 and 20 nm, respectively. The interval distance of the nanochannels was 15 μm. The concentration of the KCl solution used was 0.1 mM. The current measurement experiments were carried out under DC power (applied by an Ag electrode) with an increment of 0.5 V for 3 s duration. As shown in the inset diagram in Fig. 2(b), the Ag electrodes were put into the reservoirs (H_2_ and H_4_) and the hydrodynamic flow was induced into the H_2_ reservoir. Fig. 3(a) shows a schematic diagram of the flow in a nanofluidic chip. There is a small region at the end of the nanochannels, where it is affected slightly by the inflow without applying a voltage. The ICP phenomenon occurs at this region under the influence of the applied voltages when the inflow is 0 nL min^−1^. In addition, the ICP region would increase without an induced inflow. The conductance of the nanofluidic chips depends on the resistance of the nanochannel and the ICP region because of the lower ion concentration in them. The induced flow could control the increase in the ICP region and more ions were taken by the inflow to the ICP region, which improved the ion concentration in this area. Fig. 3(b) presents the electric current measurement results for the hydrodynamic inflow of KCl from 0 to 70 nL min^−1^. To show the results clearly, the inset in Fig. 3(b) displays the measured results for hydrodynamic inflow from 0 to 35 nL min^−1^. It is noticeable from Fig. 3(b) that the initial limiting currents for no and lower inflow were smaller than those for the inflow that from 40 to 60 nL min^−1^, and there is almost no limiting current when the inflows are 65 and 70 nL min^−1^. In addition, the electric current increased obviously with the increasing inflow when the inflows are in the range from 40 to 60 nL min^−1^. However, the electric currents collapse onto each other when the inflow are 65 and 70 nL min^−1^. The width of the main microchannel was 80 μm, and the distance between the nanochannel and the boundaries of the microchannel was 20 μm. The hydrodynamic convection was mainly generated at the joint between the main and side channels at lower inflow rates (25–35 nL min^−1^) and the effect of the inflow on the ICP region was relatively slight in this circumstance. Thus, the change in the *I*–*V* curve was not clear. However, when the inflow reached 40 nL min^−1^, the hydrodynamic convection was generated at the end of the nanochannels and the ICP area was smaller compared with a lower flow rates, thus, which leads to the electric current increasing promptly. When the inflow reached to 65 nL min^−1^, the effect of ICP is almost eliminated due to the relatively large inflow. Thus, the electric current increases linearly with the applied voltage increasing in these scenarios. Results show that the conductance of the nanofluidic chip can be improved by inducing hydrodynamic convection at the ICP region. The present work may prompt the application of nanofluidic chips in energy-harvesting systems due to the weakened influence of the limiting current behavior.

### Electrical detection of enzymatic reaction in single nanochannel

Enzymatic reactions play a vital role in a variety of biological processes, and the abnormalities in specific enzyme reactions commonly result in disease. Presently, use of a fluorogenic/chromogenic-labeled substrate is a common method to monitor enzyme reactions. However, these approaches are expensive and time-consuming.^37^ The nanochannels contained in nanofluidic chips are at the scale from several to tens of nanometers. Thus, the reactants are confined within a relatively small space, leading to advantages such as high sensitivity, lower cost, and quick response times. The label-free detection of enzyme reactions based on a nanofluidic device has huge potential for the field of bioengineering. The enzymatic reaction between trypsin and PLL was selected in this work to demonstrate the application of a nanofluidic chip to label-free detection by monitoring the electric current.

**Fig. 4:**
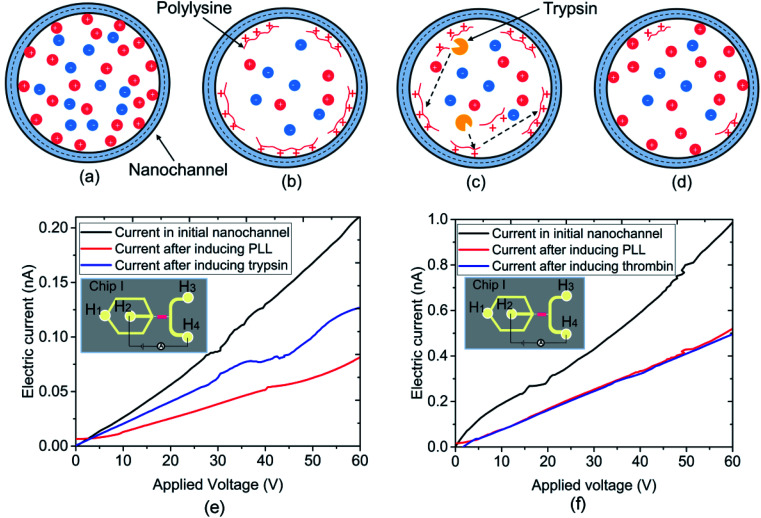
Schematic diagram of the experimental processes for the reaction between trypsin and polylysine. (a) Original PDMS nanochannel surface, (b) PLL coating on the nanochannel surface, (c) trypsin reacted with PLL, and (d) the cleaved substrate and trypsin were removed. Electric current measurement results for reaction between PLL and (e) trypsin and (f) thrombin.

Trypsin, a serine proteolytic enzyme extracted from the pancreas of cattle, sheep, and pigs, acts as a digestive enzyme in vertebrates. It mainly cleaves the peptide chain on the carboxyl side of amino acids lysine and arginine, and the process is commonly referred to as trypsin proteolysis. The PLL can be cleaved by trypsin into bi- or tri-lysine segments. Fig. 4(a)–(d) demonstrate the experimental processes for the reaction between trypsin and PLL. The conductance of the initial nanochannel in DI water was measured first. Then, the nanofluidic device was filled with PLL (concentration of 100 mg L^−1^), and it was immersed for 1 h to make the PLL absorb onto the nanochannel surface. The pH value of the PLL solution is 6.9, which is lower than the PLL isoelectric point, thus the PLLs are positively charged. The system was then rinsed using DI water for 5 min to remove any residual PLL. The surface of the PDMS nanochannel was negatively charged, meaning the positively charged PLLs could be absorbed onto the nanochannel surface automatically, as shown in Fig. 4(b). The conductance of the nanofluidic device was measured again subsequently. The trypsin with a concentration of 10^5^ ng mL^−1^ in buffer solution was then induced into the nanofluidic device for a given reaction time. As shown in Fig. 4(c), the PLL was digested by trypsin into tri- or di-lysines and desorbed from the surface of the nanochannel. Subsequently, the nanofluidic device was washed with buffer solution for 5 min to remove the trypsin, followed by rinsing with DI water before the final measurement of the nanochannel conductance. To investigate the specificity of the reaction between trypsin and PLL, another serine protease (thrombin) was selected instead of trypsin to react with PLL in this work. Thrombin is involved in the blood coagulation cascade and it shows very little activity toward PLL. The concentration of the thrombin solution used herein was 10^5^ ng mL^−1^.

Fig. 4(e) presents the electric current measurement results for the reaction between trypsin and PLL. The experiments were carried out on chip I, where the width and depth of the nanochannel were 248 and 19 nm, respectively. The current was measured by the KickStart software with an increment of 0.5 V for 3 s in duration. It is observable that the electric current obtained at the initial nanochannel in DI water is the highest. However, the electric current was lowest when the PLL was induced into the nanofluidic device. In addition, it increased slightly after the trypsin was added into the nanochannel. The change in conductivity was caused by the variation of the surface charge density of the nanochannel when conducting the experiments. The silane groups on the nanochannel surface dissociated first in DI water, which led to the surface charge density of the nanochannel increasing. According to eqn (1), the conductance of the nanochannel is influenced by the surface charge density. Thus, the conductivity of the initial nanochannel was highest in this circumstance. However, the positively charged PLL was absorbed onto the negatively charged nanochannel surface after being induced into the nanofluidic device. The nanochannel surface, thus, changed to neutral or slightly positively charged because of the absorption of PLL.^10,38^ The conductivity of the nanofluidic device clearly decreased with the variation in the surface charge density, as shown in Fig. 4(e). However, the original PDMS nanochannel surface was reexposed to the solution after the trypsin was induced into the nanochannel and it cleaved the PLL into bi- or tri-lysine segments from the nanochannel surface. Hence, the conductivity of the nanochannel increased again. Fig. 4(f) shows the electric current measurement results for the reaction between thrombin and PLL. It is noticeable in Fig. 4(f) that the electric current in the initial nanochannel is highest; however, the current showed almost no difference between before and after the thrombin was induced into the nanofluidic device. Thrombin has little activity towards PLL and the PLL cannot be cleaved by it in the nanochannel, which led to the surface charge density showing no change. Consequently, the conductivity of the nanochannel after inducing the thrombin had no variation. The experimental results, as indicated by the change in the detected electric current, prove the reaction specificity between trypsin and PLL. Furthermore, the electric current change in the nanofluidic device could be used as an indicator to detect enzymatic activity in label-free detection.

## Conclusions

Nanofluidic chips with different numbers of nanochannels were fabricated based on a commercial AFM system using the single scratching approach. Two applications for the prepared nanofluidic chips were demonstrated, which included detection of an enzymatic reaction and electrical characterization, such as the effect of dynamic convection. The conductance of the nanofluidic chip was affected by the number of nanochannels and the solution concentration. Results show that the conductance for nanofluidic chips with three nanochannels was better than for those with a single nanochannel when the nanochannels have identical features and dimensions. The higher resistance of the nanofluidic chip with a single nanochannel was considered as the influencing factor. Furthermore, the conductance of the nanofluidic chips, which is governed by the bulk concentration at high ionic concentrations, going up with the solution concentration increasing. In addition, the hydrodynamic convection, generated from the induced inflow and at the end of the nanochannel, had an influence on the conductance of the nanofluidic devices. The change in the obtained *I*–*V* measurement curve was slight when the inflow was lower than 35 nL min^−1^. However, the conductance clearly increased when the inflow reached 40 nL min^−1^. This is likely because more ions were carried to the ICP region when the inflow was greater. Finally, the reaction specificity between trypsin and PLL was proven by means of the electrical detection of the enzymatic reaction conducted in a single nanochannel. This means that the electric current change in a nanofluidic device may be used as a label-free indicator to detect enzymatic activity.

## Author contributions

Yongda Yan and Yanquan Geng carried out the design. Jiqiang Wang completed most of the experiments and drafted the manuscript. Leyi Chen participated in the experiments. Yang Gan and Shunyu Chang assisted with the optimization and proofed the manuscript. All authors read and approved the final manuscript.

## Conflicts of interest

There are no conflicts to declare.

## Supplementary Material
